# Hu-Zhang-Qing-Mai-Yin Inhibits Proliferation of Human Retinal Capillary Endothelial Cells Exposed to High Glucose

**DOI:** 10.3389/fphar.2021.732655

**Published:** 2021-08-06

**Authors:** Yuan-Yuan Yu, Qiu-Ping Liu, Meng-Ting Li, Pei An, Yu-Ying Chen, Xin Luan, Chao Lv, Hong Zhang

**Affiliations:** ^1^ Institute of Interdisciplinary Integrative Medicine Research, Shanghai University of Traditional Chinese Medicine, Shanghai, China; ^2^ Department of Neurology, Yueyang Integrated Traditional Chinese and Western Medicine Hospital, Shanghai University of Traditional Chinese Medicine, Shanghai, China

**Keywords:** diabetic retinopathy, Chinese medicine formula, human retinal capillary endothelial cells, proliferation, apoptosis

## Abstract

Background: Diabetic retinopathy (DR) is one of the serious complications of diabetes and an important cause of blindness. Despite much research on the pathogenesis of DR, there is still a lack of safe and effective treatment methods. Hu-zhang-qing-mai-yin (HZQMY), a Chinese medicine formula, has been clinically used in the safe and effective treatment of DR for many years. However, the systematic pharmacological research is lacking. The aim of this study was to evaluate the anti-DR effects of HZQMY and explore the possible mechanism involved. Methods: The constituents of HZQMY were analyzed by LC-MS/MS. DR model was established by high glucose simulation on human retinal capillary endothelial cells (HRCECs) *in vitro*. The cell viability, cell proliferation, cell apoptosis, and tube formation were assessed. Subsequently the related mechanisms were analyzed by assays for JC-1 mitochondrial membrane potential (MMP), intracellular ROS, ATP, western blot and proteomics. Results: 27 main chemical components contained in HZQMY were identified. HZQMY significantly inhibited the viability and proliferation of HRCECs exposed to high glucose, and promoted the apoptosis. In addition, HZQMY also boosted the release of ROS and suppressed tube formation of HRCECs under high glucose exposure. Meanwhile, HRCECs treated with high glucose released more ROS than normal cells, which could be markedly inhibited by HZQMY in a dose-dependent manner. Additionally, western blot assay indicated that HZQMY increased the expression of proteins related to the P38 signaling pathway and inhibited nuclear factor kappa-B (NF-κB) pathway. Proteomic analysis predicted that HSPA4, MAPK3, ENO1, EEF2 and ERPS may be the candidate targets of HZQMY in HRCECs. Conclusions: HZQMY inhibited the proliferation and promoted the Mitochondria related apoptosis of HRCECs exposed to high glucose possibly through regulating P38 and NF-κB signaling pathway.

## Introduction

Diabetes is one of the most common chronic metabolic diseases, with an estimated 592 million diabetics worldwide by 2035 (Duh et al., 2017). Diabetic retinopathy (DR) is a highly specific neurovascular complication of type 1 and type 2 diabetes, which is one of the most common complications of diabetes and affects about one third of diabetic patients (Wong and Sabanayagam, 2020). The prevalence of diabetes among adults (20–79 years old) has risen to 8.8% and diabetic patients are more prone to suffer from cataract, glaucoma and other eye diseases (Bommer et al., 2017). Diabetes has a profound impact on multiple organ systems, but visual impairment and even blindness caused by DR may be one of the diseases that have the greatest impact on patient’s quality of life (Antonetti et al., 2021). At present, the main treatment methods for DR include laser therapy, intravitreal injection of steroid hormones or taking anti-VEGF drugs, oral administration of calcium hydroxybenzene sulfonate, etc., but there are problems of ineffective or accompanied by a large number of side effects (Ihnat et al., 2007). Therefore, it is an urgent need for finding the therapy with good safety and effectivity.

In recent years, clinical studies have shown that the application of Chinese medicine in the prevention and treatment of DR has significant advantages due to the definite clinical efficacy (Xiao and Luo, 2018; Zhang et al., 2018). Based on modern pharmacology and clinical trials, Chinese medicines have the advantages of multi-target and multi-channel in the treatment of DR (Behl and Kotwani, 2017; He et al., 2016; Pang et al., 2015; Zhang et al., 2018). Clinical trials have shown that Chinese medicines can promote blood microcirculation, improve vascular endothelial function, protect the blood-retinal barrier, and inhibit oxidation and inflammation (He et al., 2016; Behl and Kotwani, 2017; Zhang et al., 2018).

Hu-zhang-qing-mai-yin (HZQMY) is an empirical Chinese medicine prescription produced by combining the theory of traditional Chinese theory with clinical experience, which is composed of root of *Polygonum cuspidatum* Sieb. et Zucc., fruit of *Forsythia suspensa,* whole herb of *Sedum sarmentosum* Bunge, whole herb of *Siegesbeckia orientalis* L., rattan stem of *Spatholobus suberectus* Dunn, root of *Glycyrrhiza uralensis* Fisch, root of *Stragalus membranaceus* (Fisch.) Bunge and root of *Ligusticum chuanxiong* hort. The formula plays the function of clearing heat and detoxification, promoting blood circulation and removing blood stasis. However, its active components and mechanism of action are still unclear, and systematic pharmacological studies on the treatment of DR are lacking.

In our study, the main ingredients of HZQMY were identified by HPLC-Q-TOF-MS, its anti-DR effects were evaluated and the mechanism of action was explored *in vitro*, so as to provide reference for the prevention and treatment of DR.

## Materials and Methods

### Cells and Regents

Human retinal capillary endothelial cells (HRCECs) were acquired from the Cell Bank of Shanghai Academy of Chinese Sciences (Shanghai, China). Dulbecco’s modified eagle medium (DMEM) was purchased from HyClone (Logan, United States), fetal bovine serum (FBS) was bought from GIBCO (Grand Island, United States). Cell Counting Kit-8 (CCK-8) and PBS were obtained from meilun biotechnology co., Ltd. (Dalian, China). ATP Assay Kit, Mitochondrial membrane potential kit and ROS assay kit were purchased from Beyotime Biotechnology (Shanghai, China). Apoptosis Detection Kit and cycle detection kit were provided by KeyGen Biotechnology co., Ltd. (Nanjing, China). Matrigel was provided by Corning (New York, United States). Primary antibody of p-NF-κB, NF-κB, p-P38, P38, BCL-XL, BCL-2 and GAPDH were bought from Cell Signaling Technology (Danvers, United States).

### Preparation of Hu-Zhang-Qing-Mai-Yin Extract

Root of *Polygonum cuspidatum* Sieb. et Zucc., fruit of *Forsythia suspensa*, whole plant of *Sedum sarmentosum* Bunge, whole plant of *Siegesbeckia orientalis* L., rattan stem of *Spatholobus suberectus* Dunn, root of *Glycyrrhiza uralensis* Fisch, root of *Stragalus membranaceus* (Fisch.) Bunge and root of *Ligusticum chuanxiong* hort. were provided by the ophthalmology Department of Longhua Hospital Affiliated to Shanghai University of Traditional Chinese Medicine (Table 1). These Chinese herbal medicines were put into the decocting pot, and extracted with 10 times volume pure water for 1 hour, two times in total. The decocting liquid were concentrated with a rotating evaporator after filtering with a 4-layer gauze. After that, the liquid was further pre-freezed at −50°C for 5 h for a vacuum dry in the material tray of the freeze-dryer. The dryer was heated up and finally fixed to −40°C for lyophilized 72 h to make a lyophilized powder which was stored in a refrigerator at −20°C for later use. The stock solution of the HZQMY extract was prepared with first grade pure water to the concentration of 0.1 g/ml, and then it was filtered and sterilized by 0.22 μm microporous membrane. For temporary use, the medium containing 10% FBS was diluted to the working concentrations.

**TABLE 1:** Components of HZQMY.

Plant name	Medicinal part	Dosage (g)
*Polygonum cuspidatum* Sieb. et Zucc	Root	18
*Forsythia suspensa*	Fruit	18
*Sedum sarmentosum* Bunge	Whole plant	30
*Siegesbeckia orientalis* L	Whole plant	18
*Spatholobus suberectus* Dunn	Rattan stem	15
*Glycyrrhiza uralensis* Fisch	Root	9
*Stragalus membranaceus* (Fisch.) Bunge	Root	15
*Ligusticum chuanxiong* hort	Root	18

### LC-MS/MS Analysis

1.0 g of HZQMY extract was put into a 25 ml volumetric flask, and then 50% methanol was added to scale, ultrasonic treatment for 30 min, cooling, 50% methanol to make up the amount of loss reduction, fully mixed, filtrated with a 0.45 μm microporous membrane to obtain the test solution, and 10 μL was taken for mass spectrometry detection.

Agilent 6530 four-stage rod-time-of-flight mass spectrometry (Q-TOF-MS) system was performed, chromatographic separations were carried out on an ACQUITY UPLC HSS T3 (2.1 × 150 mm, 1.7 μm) column at 25°C and the drying gas (N_2_) was 13 L/min. Gradient elution with mobile phase A (0.1 formic acid water), mobile phase B (acetonitrile): 99% A in 0–2 min; from 99 to 34% A in 2–20 min; from 34 to 5% A in 20–28 min. The mass spectrometry was performed in positive and negative ion scanning patter with a scanning range of 100–1700 m/z and a capillary voltage of 4000 V. The intercepting cone voltage is 60 V.

### Cell Culture

HRCECs were cultured in DMEM containing 10% FBS and 1% penicillin-streptomycin at 37°C and 5% CO_2_. DR model was established by induction of HRCECs with high glucose *in vitro* (Liu et al., 2020). The cells were divided into normal group (cells were cultured in normal-glucose (5.5 mM) media, NG), high glucose model group (cells were cultured in high-glucose (35 mM) media, HG), and HZQMY different dose groups. Except normal group, all the other groups of cells were given 35 mM glucose to imitate high-glucose environment. Different concentrations of HZQMY extract were used to treat the cells. Cells were collected for subsequent experiments following treatment with HZQMY extract for 24 h or 48 h.

### CCK-8 Assay

HRCECs were seeded into 96-well plates (5 × 10^3^ cells/well) for 24 h in a 5% CO_2_ incubator at 37°C, then different concentrations of HZQMY extract (10, 25, 50, 100, 150 μg/ml) were added in 96-well plates for 24 and 48 h. Discarding the old medium, 100 μL of 10% CCK-8 kit was added into each well for incubation at 37°C for 30 min. OD value was detected at 450 nm with a microplate analyzer. Cell survival rate (%) = (OD value of experimental group–OD value of blank group)/(OD value of control group–OD value of blank group) ×100%.

### Cell Colony Formation Assay

HRCECs were seeded into 6-well plates (600 cells/well) for 24 h in a 5% CO_2_ incubator at 37°C, then added with different concentrations of the extract and cultured for 8 days. The cells were fixed with 4% paraformaldehyde for 30 min and stained with crystal violet for 15 min. Clone formation rate (%) = (amount of clones/number of inoculated cells) ×100%.

### Cell Cycle Analysis

HRCECs were seeded into 6-well plates (5 × 10^5^ cells/well) for 24 h in a 5% CO_2_ incubator at 37°C, then each group was added with different concentrations of the extract for 24 h culture. Cells were harvested, 1 ml of precooled 70% ethanol was added to mix well, and fixed at 4°C for 2 h. The cells were collected, mixed with 0.5 ml PI into the sample tube, and incubated in the dark for 30 min. The red fluorescence was detected by flow cytometry at 488 nm, and the light scattering was recorded at the same time. The Beckman flow cytometer was used for detection.

### Tube Formation Assay

60 µL of the dissolved matrigel (9–12 mg/ml) was added in a well in 96-well plate and then placed in the incubator at 37°C for 1 h. HRCECs were cultured under different concentrations of HZQMY with or without HG. Subsequently, 50 μL of cell suspension was collected and added into the pre-solidified matrigel for 8 h in the 37°C incubator. The tube formation was observed under an optical microscope, and photographed. ImageJ was used to calculate the number of lumens and the length of tubules.

### Cell Nuclear Staining

HRCECs were seeded into 24-well plates (5 × 10^4^ cells/well) for 24 h in a 5% CO_2_ incubator at 37°C, then each group was added with different concentrations of the extract for 24 h culture. Subsequently, the cells were washed by PBS three times, 150 µL of 4% paraformaldehyde was added to each well and fixed at room temperature for 30 min, and remove the fixing solution, add 150 µL of 0.1% Triton X-100 for 10 min. Finally, 150 µL DAPI solution was added in each well for incubation at room temperature for 10 min in dark. The samples were photographed by Operetta CLS high-content analysis system.

### Apoptosis Assay

HRCECs were seeded into 6-well plates (5 × 10^5^ cells/well) for 24 h in a 5% CO_2_ incubator at 37°C, then each group was added with different concentrations of drugs for 24 h culture. After that, the cells were digested with trypsin without Ethylene Diamine Tetraacetic Acid (EDTA) and centrifuged with a 15 ml centrifuge tube at 2000 rpm for 10 min at 4°C, and the supernatant was discarded. 100 μL 1 × FITC binding solution was added to mix well, then 5 μL FITC dye was added for 10 min incubation at room temperature in dark. Last, 5 μL PI dye was added for 5 min incubation in dark. The Beckman flow cytometer was used for detection.

### Intracellular ROS Detection

HRCECs were seeded into a 96-well plate (1 × 10^5^ cells/well) for 24 h in a 5% CO_2_ incubator at 37°C, then each group was added with different concentrations of the extract for 6 h. DCFH-DA was added for 1 h incubation in a 37°C cell incubator. The Beckman flow cytometer was used for detection.

### ATP Detection

ATP concentration was detected by the ATP Assay Kit. HRCECs were seeded into 6-well plates (5 × 10^5^ cells/well) for 12 h in a 5% CO_2_ incubator at 37°C, then added with different concentrations of HZQMY extract to culture for 24 h. Cells were lysed to isolate total protein, then centrifuged at 12,000 g and 4°C for 10 min. Next, 20 µL sample or standard solution were added to 100 µL ATP detection solution, mixed, and luminescence was measured with a multifunctional enzyme plate analyzer.

### JC-1 Mitochondrial Membrane Potential Assay

HRCECs were seeded into 24-well plates (5 × 10^4^ cells/well) for 24 h in a 5% CO_2_ incubator at 37°C, then added with different concentrations of the extract for 24 h culture. Apoptotic cells should show green fluorescence after JC-1 staining, while normal cells show red fluorescence. The relative ratio of red-green fluorescence is often used to measure the proportion of mitochondrial depolarization, which was used as one of the early detection indicators of cell apoptosis. The fluorescence quantification was carried out using ImageJ software.

### Western Blot Assay

HRCECs were seeded into 6-well plates (5 × 10^5^ cells/well) for 24 h in a 5% CO_2_ incubator at 37°C, then each group was added with different concentrations of the extract for 24 h culture. Cells were collected and total proteins were extracted from HRCECs using RIPA lysate and PMSF (RIPA: PMSF = 100:1). Protein concentration was measured by using the BCA Protein Concentration Assay Kit. The proteins were separated by 10% SDS-polyacrylamide electrophoresis and transferred to PVDF membrane, which was then sealed with QuickBlock™ Blocking Buffer at room temperature for 1 h. After that, the membrane was incubated with the primary antibody overnight at 4°C, followed by washing 3 times with TBST, and the rabbit/mouse antibody was incubated at room temperature for 1 h. Then the membrane was washed 3 times with TBST. Finally, the gray values of the protein bands were detected and photographed by Chemi Scope Mini, and GAPDH was used as internal reference.

### TMT Quantitative Proteomic Analysis

HRCECs (cultured with 35 mM glucose) were seeded into 100 mm culture dishes and 100 µg/ml HZQMY extract was added until 60% confluent. After 24 h, cells were harvested and flash-frozen in liquid nitrogen. Identification and analysis were processed by Ouyi Biotechnology Co., Ltd. (Shanghai, China). The proteins were quantified by iTRAQ labeling, and based on UniProt, KEGG, go, KOG/cog databases, the annotated information of the identified proteins was extracted to mine the protein functions. Proteins with quantification changes >1.5 and *p* value <0.05 were considered as differentially expressed proteins. After the differentially expressed proteins were obtained, GO/KEGG enrichment analysis were performed to describe the functions of these proteins, and the involved interaction network analysis was also executed by using STRING database.

### Statistical Analysis

All data were analyzed by SPSS 22.0 statistical software, and the results were expressed as mean ± standard deviation. The comparison of the mean between two groups was performed by *t*-test, and the comparison of the mean between multiple groups was carried out by one-way analysis of variance. *p* < 0.05 was considered statistically significant.

## Results

### Identification of the Components of Hu-Zhang-Qing-Mai-Yin

**FIGURE 1:**
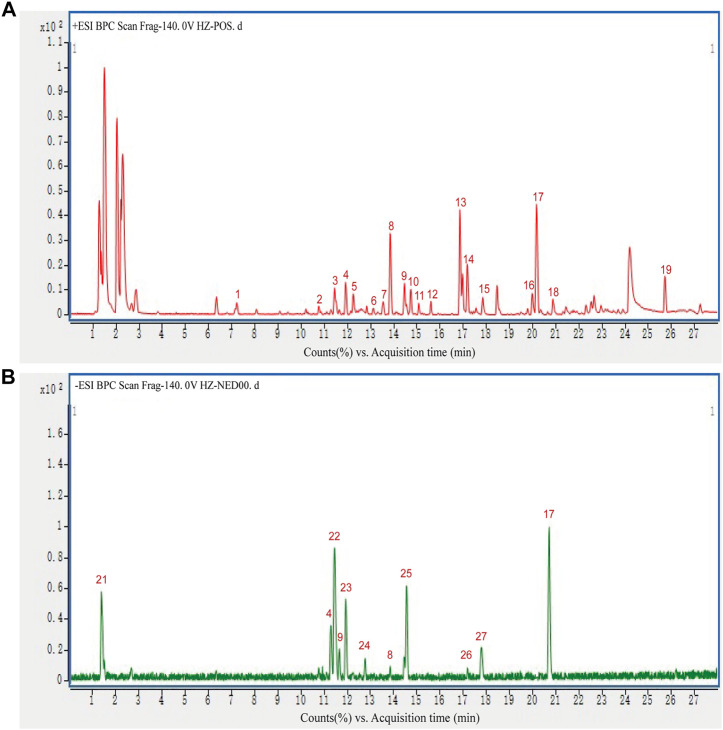
HPLC-Q-TOF-MS chromatogram of 27 constituents in positive and negative mode.

HPLC-Q-TOF-MS was used to analyze the constituents of HZQMY, and a total of 27 compounds were identified by comparing databases and literatures, which are Forsythoside E (1), Heteroclitin D (2), Quercetin (3), Forsythoside (4), Pinoresinol 4-O-β-D-glucopyranoside (5), Quercitrin (6), Forsyithin (7), Arctiin (8), Polydatin (9), Liquiritin (10), Apigenin (11), Isorhamnetin (12), Calycosin-7-O-glucoside (13), Kaempferol (14), Afromosin (RG) (15), (-)-Catechin hydrate (16), Genistein (17), Eleutheroside A (18), *trans*-Anethole (19), Oleanic acid (20), Quercetin dihydrate (21), Physcion 8-β-D-glucoside (22), Hyperoside (23), 3-Hydroxy-4-methoxybenzoic acid (24), Luteolin (25), Formononetin (26) and Prunetin (27). The chromatogram of the compounds obtained is shown in Figure 1.

### Hu-Zhang-Qing-Mai-Yin Inhibited Cell Viability and Proliferation

**FIGURE 2:**
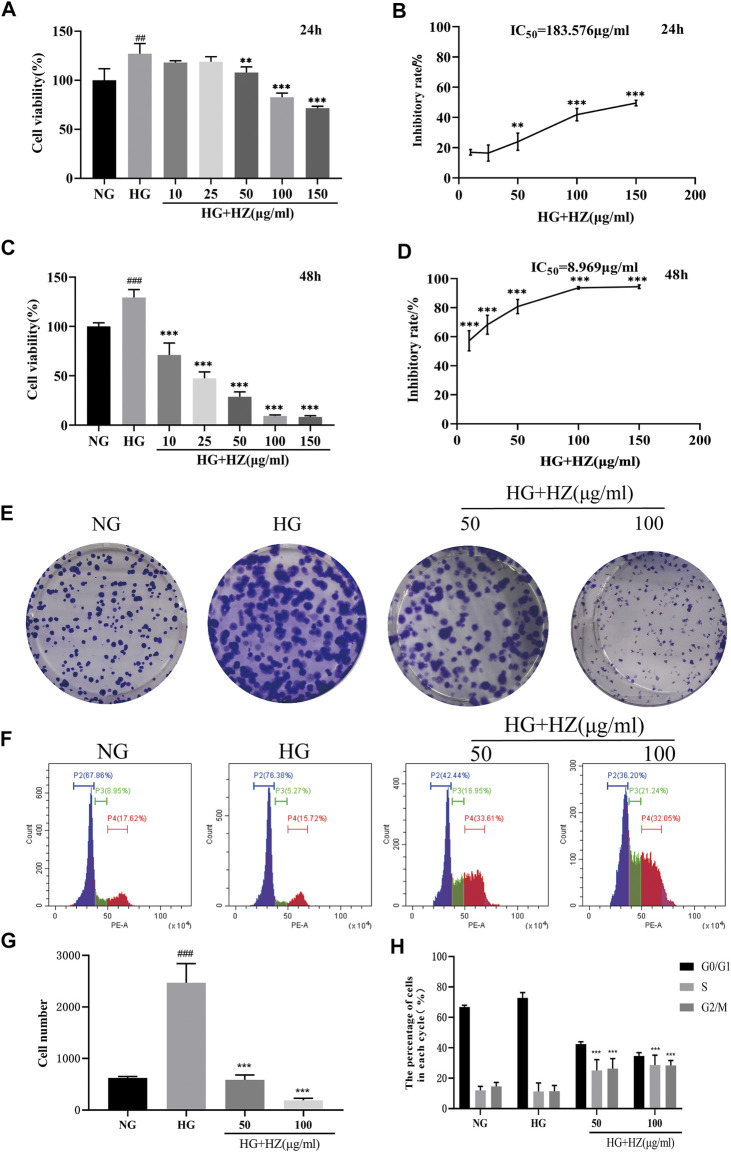
Effects of HZQMY on the viability and proliferation of HRCEC cells exposed to high glucose. Cell viability **(A**, **C)** and inhibitory rate of cell proliferation **(B**, **D)** induced by various concentrations of HZQMY for 24 and 48 h, respectively. **(E)** HRCECs were cultured with NG, HG and HG-HZ, respectively, for 8 days **(F)** Cell cycle distribution was detected by flow cytometry. **(G)** The number of cell colonies was analyzed. **(H)** The percentage of cells in each phase was analyzed. ^##^ and ^###^ indicate *p* < 0.01 and *p* < 0.001, respectively, compared with NG group. ^**^ and ^***^indicate *p* < 0.01 and *p* < 0.001, respectively, compared with HG group. HZ: HZQMY.

As shown in Figure 2, when compared with NG group (5.5 mmol/L), the cell viability significantly increased in HG group (35 mmol/L). However, 50–150 μg/ml HZQMY significantly reduced the cell viability increased by high glucose after 24 h incubation (Figures 2A,B), and the inhibitory effect was more pronounced at 48 h (Figures 2C,D). These results suggested that high glucose can improve cell viability, while HZQMY can reverse the promotion effect in a time and dose-dependent manner.

To test the proliferation effect of HZQMY on HRCECs, cell colony formation and cycle assay were performed. Figures 2E,G show that high concentration of glucose can promote cell proliferation, while HZQMY could reverse this effect in a dose-dependent manner. Decreased cell proliferation is always associated with changes in the phase of the cell cycle, and the flow cytometry analysis displayed that the G_2_/M phase accounted for a higher proportion in the HZQMY group than in the HG group (Figures 2F,H). The results suggested that HZQMY inhibits cell proliferation by arresting the cell cycle in the G_2_/M phase.

### Hu-Zhang-Qing-Mai-Yin Decreased the Tube Formation

**FIGURE 3:**
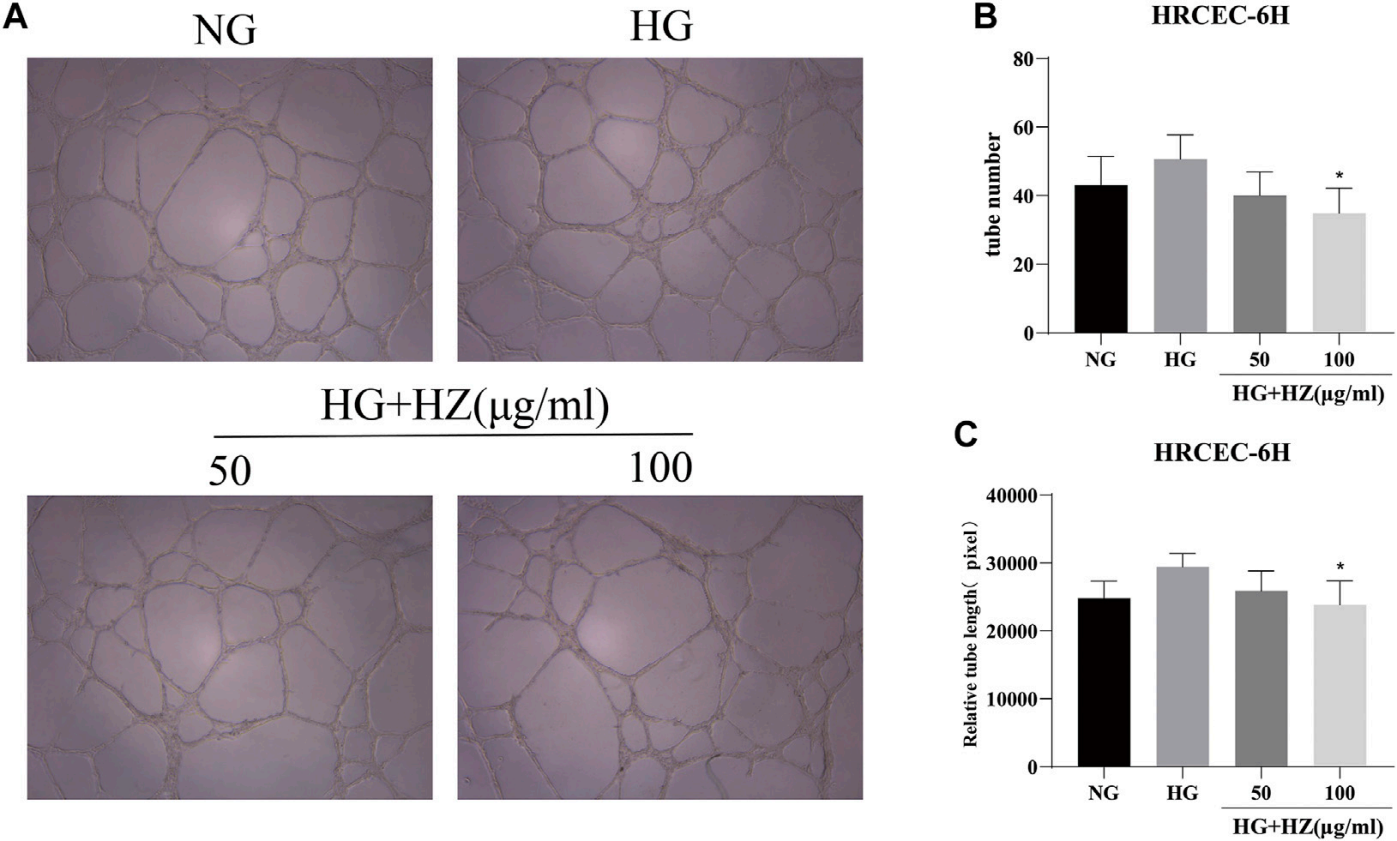
Tube formation assay of HZQMY. **(A)** Tubulogenesis simulates angiogenesis *in vivo*. Magnification, ×40. **(B)** The number and length **(C)** of tube formation were analyzed. ^*^ indicates *p* < 0.05 vs HG group. HZ: HZQMY.

Angiogenesis is a characteristic of endothelial cells. Tube formation *in vitro* was used to simulate angiogenesis *in vivo*, and the length of angiogenic branches was finally compared with ImageJ. The results (Figures 3A–C) displayed that the number of tubes and the length of tube branches were decreased after HZQMY treatment, which indicated that HZQMY can slightly inhibit the angiogenesis of HRCECs induced by high glucose.

### Hu-Zhang-Qing-Mai-Yin Facilitated Cell Apoptosis

**FIGURE 4:**
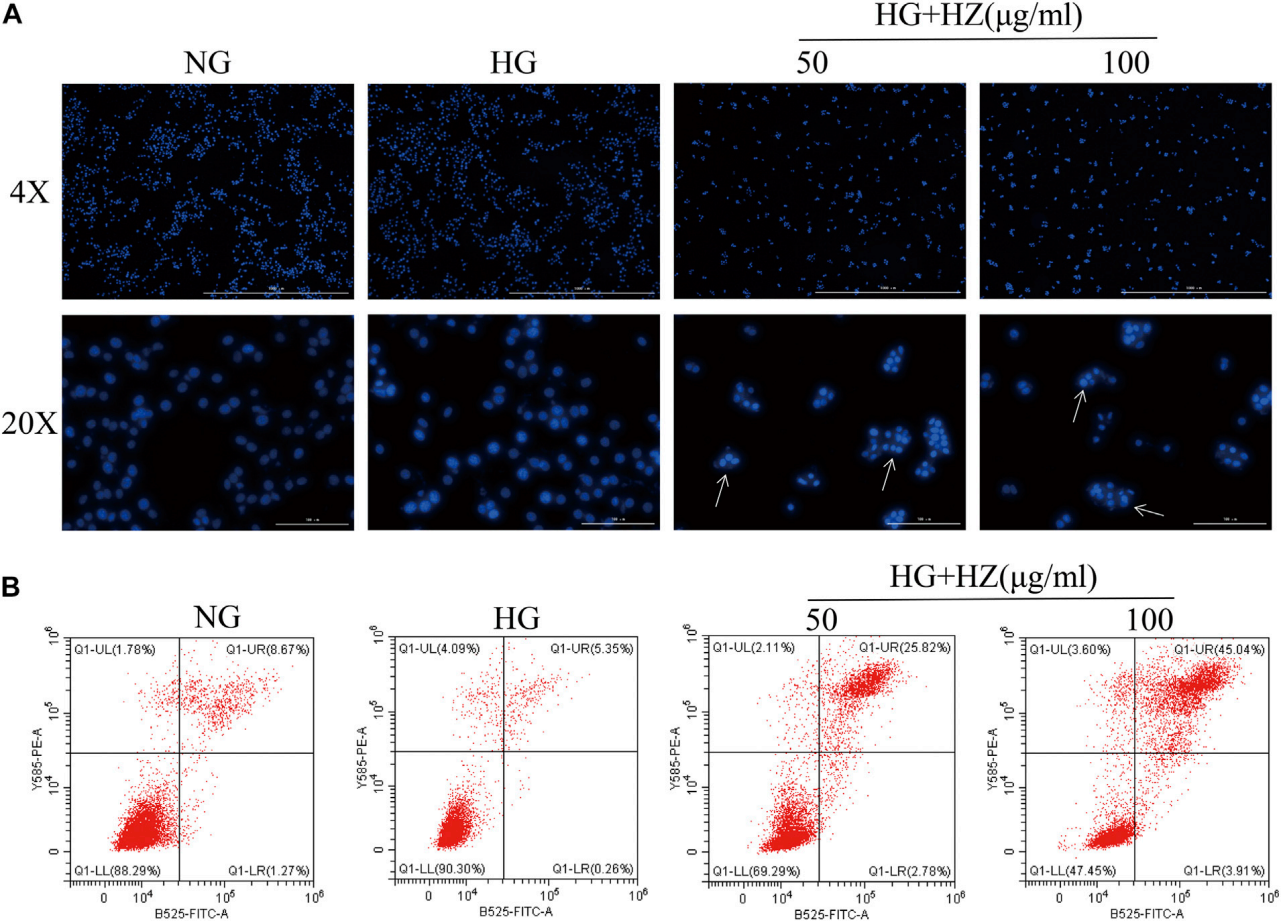
Effect of HZQMY on cell apoptosis. **(A)** Effects of HZQMY on nuclear morphology in HRCECs exposed to high glucose. **(B)** Cell apoptosis was detected by flow cytometry. Magnification, ×40 and ×200. HZ: HZQMY.

In order to determine whether the inhibitory effect of HZQMY on cell proliferation was related to the induction of cell apoptosis, the nucleus was stained with DAPI to analyze the nuclear morphology. As shown in Figure 4A, after HZQMY treatment, the number of cells decreased significantly, nuclear pyknosis and apoptotic bodies were formed in a dose-dependent manner.

Flow cytometry was used to quantitatively analyze the proportion of cells in different stages of apoptosis in each group after Annexin V and PI double staining. Annexin V is used as an indicator of early apoptosis, and PI acts as an indicator of late apoptosis. As shown in Figure 4B, with the increase of HZQMY concentration, the sum of early and late apoptosis significantly increased, with the NG group was 9.94%, the HG group was 5.61%, the HG + HZ-50 group was 28.60%, the HG + HZ-100 group was 48.95%, which suggested that HZQMY could induce cell apoptosis in a dose-dependent manner.

### Hu-Zhang-Qing-Mai-Yin Regulated ROS and ATP Levels and Induced MMP Collapse

**FIGURE 5:**
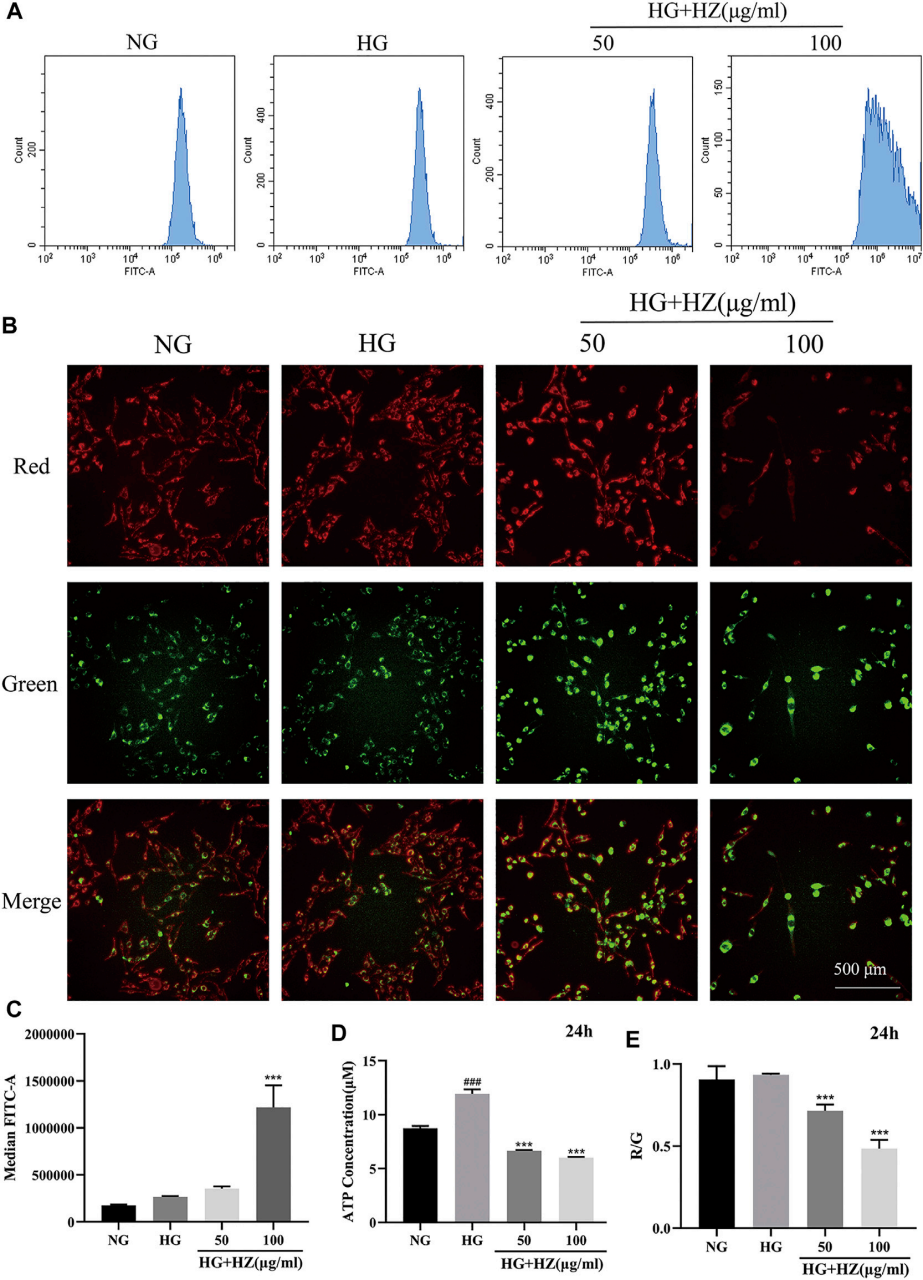
HZQMY regulated ROS and ATP levels, and promoted MMP collapse. **(A)** Cellular ROS level was detected by flow cytometry. **(B)** The red and green fluorescence of the cells was measured by high content cell imager. **(C)** The ROS level was analyzed. **(D)** Cellular ATP level was detected by ATP assay kit. **(E)** R/G represents the change of MMP. ^###^ indicates *p* < 0.001 compared with NG group. ^***^ indicates *p* < 0.001 compared with HG group. HZ: HZQMY.

Mitochondria are important organelles responsible for energy generation, which are closely related to ATP synthesis (Tang et al., 2018). What’s more, the production of ROS has also closely relation with mitochondria (Indo et al., 2007). Therefore, MMP and the levels of ROS and ATP are often used to evaluate the functional status of mitochondria. To verify whether HZQMY affected the production of ATP and ROS, the cellular ROS levels were detected by DCFH-DA and ATP content was assessed by ATP assay kit. As shown in Figures 5A,C, after treatment with 100 μg/ml HZQMY for 24 h, the production of ROS in cells increased sharply. Compared with the NG group (Figure 5D), high glucose increased ATP levels in HRCECs, while HZQMY significantly reduced its production.

JC-1 is a fluorescent probe, which can act as an indicator of early cell apoptosis, and is often used in the detection of MMP. In the NG group, the cytoplasm of the cells showed red fluorescence, indicating high MMP. While the cells are injured, MMP will decrease, the cytoplasmic red fluorescence of the cells will reduce, and the green fluorescence will increase. The ratio of red fluorescence to green fluorescence (R/G) can be used for quantitative comparison. As shown in Figure 5B, the red fluorescence significantly decreased and the green fluorescence markedly increased. The higher the concentration of HZQMY is, the lower the ratio of R/G is (Figure 5E), indicating promotive effects of HZQMY on MMP collapse.

HZQMY regulated the proteins of P38 and NF-κB signaling pathway to inhibit proliferation of HRCECs.

To explore the mechanism of inhibitory effect of HZQMY on the proliferation of HRCECs in high glucose environment, we studied its mitochondrial apoptosis pathway. The decreased MMP, ROS and ATP levels proved that mitochondrial function was impaired.

**FIGURE 6:**
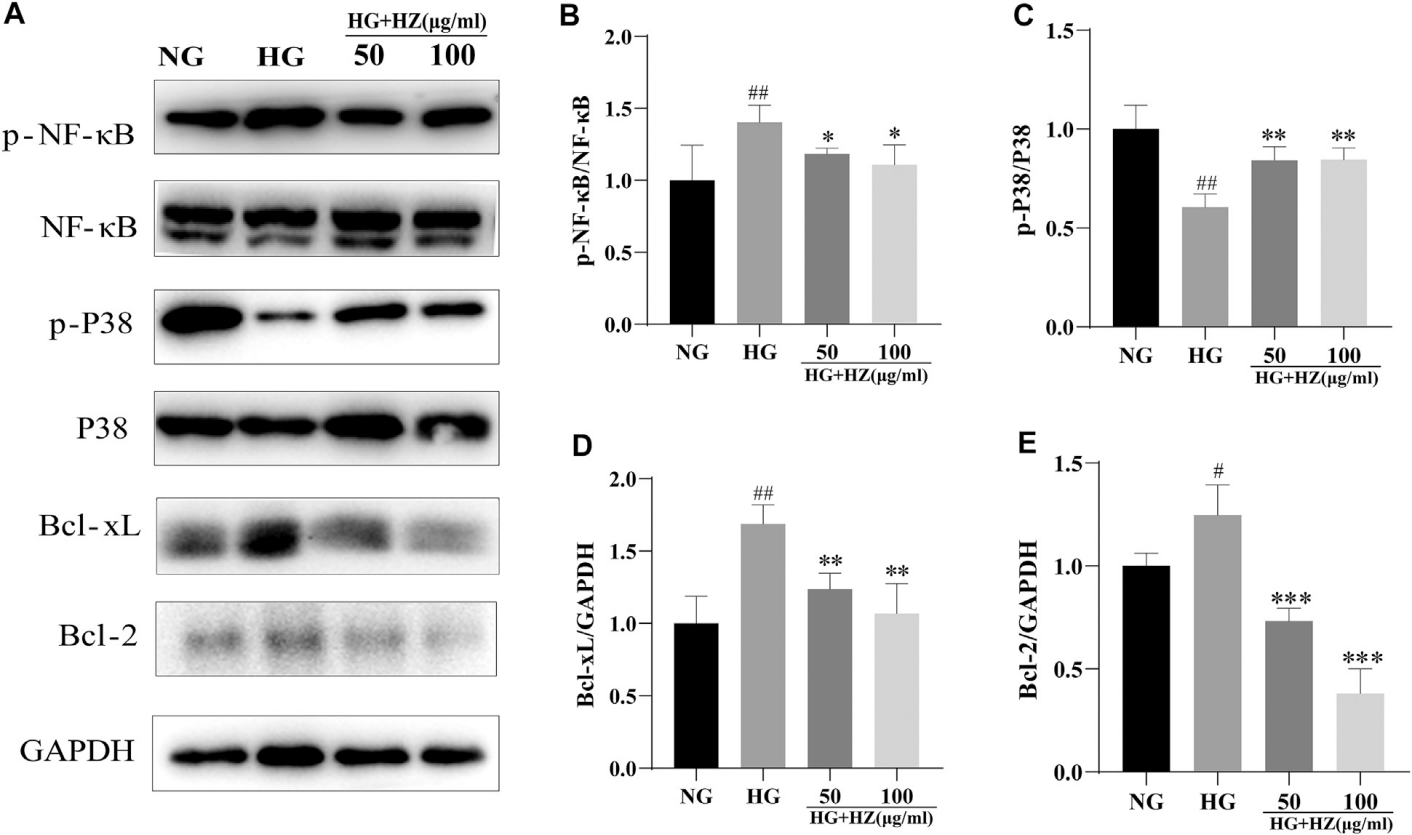
Effect of HZQMY on protein expression of P38 and NF-κB signal pathways in HRCECs. **(A)** p-NF-κB, NF-κB, p-P38, P38, BCL-XL, BCL-2 and GAPDH were measured by western blot. **(B)** The expression of p-NF-κB was analyzed. **(C)** The expression of p-P38 was analyzed. **(D)** The expression of BCL-XL was analyzed. **(E)** The expression of BCL-2 was analyzed. ^##^ and ^###^ indicate *p* < 0.01 and *p* < 0.001 compared with NG group. ^**^ and ^***^ indicate *p* < 0.01 and *p* < 0.001 compared with HG group. HZ: HZQMY.

As we all know, P38 is a classic pathway of apoptosis. In our study, P-P38 could be upregulated by HZQMY, and BCL-2 and BCL-XL could be downregulated by it, as shown in Figures 6A,C–E. Since NF-κB signal pathway plays a significant role in cell proliferation (Mu et al., 2020). Then western blot assay was used to detect the protein expression of NF-κB signaling pathway. As shown in Figures 6A,B, HZQMY downregulated the expression of p-NF-κB in HRCECs cultured with high glucose. All these results indicated that HZQMY suppressed cell proliferation possibly through regulating P38 and NF-κB signal transduction pathway.

### TMT Quantitative Proteomic Analysis

#### Enrichment and PPI Analysis of Differentially Expression Proteins

The screening conditions of differentially expression proteins were set as *p* value <0.05 and quantification fold changes >1.5. After analyze, the two groups between the HG-HRCECs group and the HZQMY-100 group have 852 differentially expression proteins, of which 122 were upregulated and 730 were downregulated. To find the potential target proteins of HZQMY on HRCECs, differentially expression proteins were compared using GO and KEGG analysis.

**FIGURE 7:**
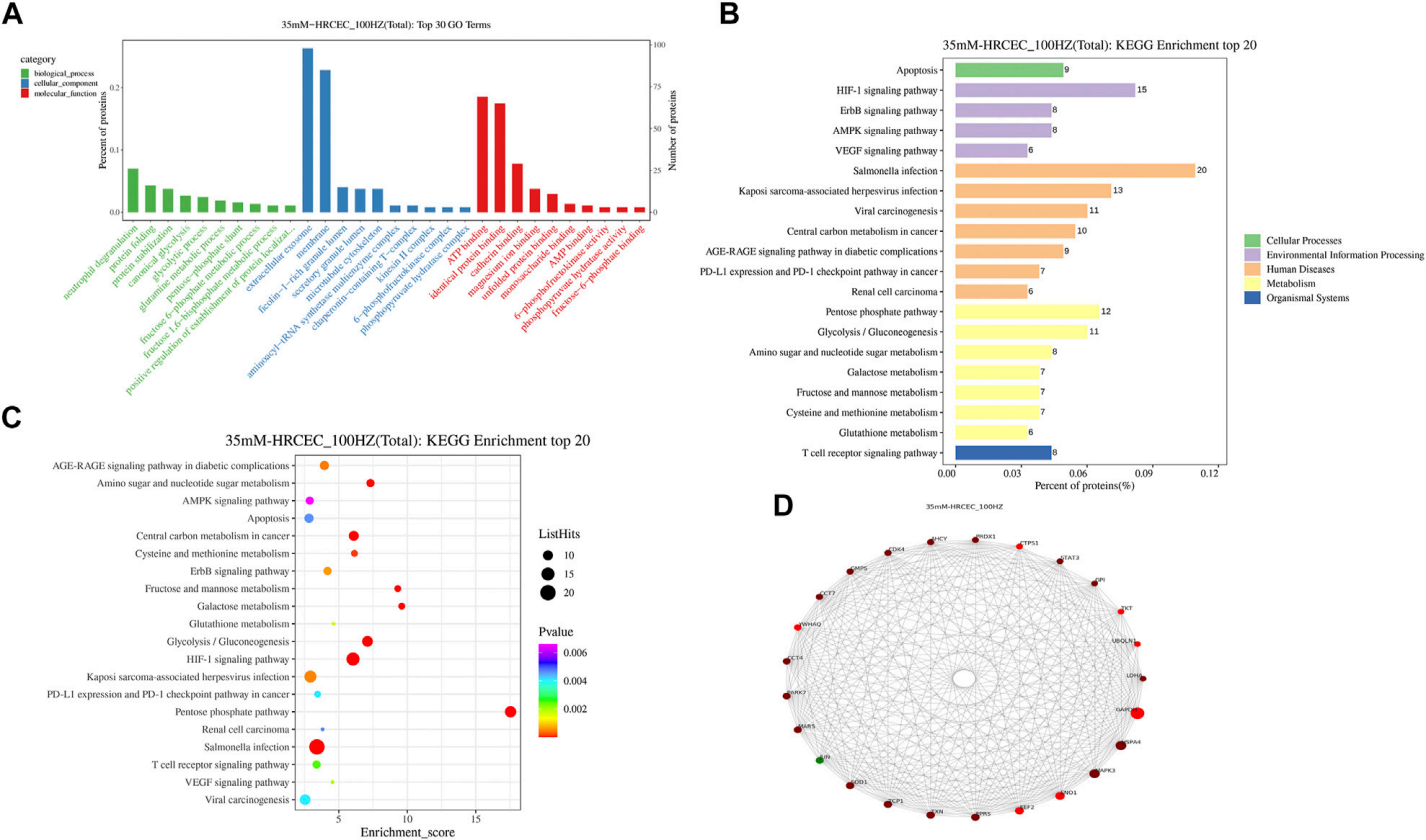
GO/KEGG/PPI analyses of differentially expression proteins. **(A)** GO enrichment of differentially expression proteins. **(B)** Differentially expression proteins distribution at KEGG Level 2. **(C)** Bubble diagram of TOP 20 KEGG enrichment. **(D)** PPI network diagram of differentially expression proteins. HZ: HZQMY.

GO analysis of differentially expression proteins mainly includes three aspects: biological processes, molecular functions and cellular component. As shown in Figure 7A, neutrophil degranulation, protein folding process were significantly enriched in the biological processes. Extracellular exosome and membrane were markedly enriched in the cellular component. ATP binding and identical protein binding were obviously enriched in the molecular function analysis.

The result of KEGG enrichment analysis showed that differentially expression proteins were significantly enriched in apoptosis, HIF-1 signaling pathway and AMPK signaling pathway (Figure 7B). The distribution of differentially expression proteins at KEGG Level 2 is shown in Figure 7C, which further reveals that the underlying mechanism of HZQMY against DR may be through apoptosis and HIF-1 signaling pathway and AMPK signaling pathway.

The String database is a database of predicted functional correlations between proteins. The species was selected in the String database for analysis of the differentially expression proteins to obtain the interaction relationship between them. Top 25 proteins with connectivity degree were selected and the interaction network diagram was drawn (Figure 7D). Top five proteins for connectivity degree are HSPA4, MAPK3, ENO1, EEF2 and ERPS.

## Discussion

Proliferative diabetic retinopathy (PDR) is the late manifestation of DR, which is one of the common serious complications of diabetic eyes, and is an important cause of vision loss and even blindness in patients (de Carlo et al., 2016). In the early stage of PDR hyperplasia, intraocular neovascularization can be controlled by total retinal laser photocoagulation, but in the late stage, PDR is often complicated with vitreous hemorrhage or neovascularization, which results in required vitrectomy. Furthermore, bleeding during this operation may affect the surgical field, prolong the operative time, increase complications and influence postoperative recovery (Abouammoh et al., 2016). In recent years, more and more studies believe that vascular endothelial growth factor (VEGF) participates in PDR neovascularization by promoting endothelial cell proliferation and migration, improving vascular permeability and other ways, leading to the progression of PDR. Therefore, it is recommended to use anti-VEGF drugs to control the progression of neovascularization ophthalmopathy (Savastano et al., 2018). However, it has been reported that conventional anti-VEGF drugs such as bevacizumab and leizumab may have certain toxic reactions while inhibiting retinal and choroidal neovascularization, affecting retinal photoreceptor function (Gross and Glassman, 2016).

Traditional Chinese medicine (TCM) in the treatment of diabetic retinopathy has its unique advantages of safety, good efficacy and less side effects. Although TCM is used as a supplement and alternative medicine in many developed countries, more than 70 percent of people take it to prevent and treat diseases in China (Chen and Lu, 2006). HZQMY has been used in clinic due to definite curative effect in DR. However, systematic pharmacological studies on the treatment of DR are lacking. In the present study, HPLC-Q-TOF-MS was used to analyze the constituents of HZQMY, 27 of which were identified. Among them, Quercetin has been reported to be effective in the treatment of type 2 diabetes (Chen et al., 2016), possibly by alleviating ferroptosis of pancreatic β cells (Li et al., 2020). Arctiin inhibits the proliferation of human retinal capillary endothelial cells induced by high glucose by regulating the ROCK 1/PTEN/PI3K/Akt/VEGF pathway (Zhou et al., 2020). Polydatin increases glucose and lipid metabolism in insulin resistant HepG 2 cells via the AMPK pathway (Hao et al., 2018). Apigenin improves diabetic nephropathy by MAPK-NF-κB-TNF-α and TGF-β1-MAPK-fibronectin pathways (Malik et al., 2017). Isorhamnetin, genistein, oleanic acid, hyperoside, luteolin and formononetin have the effect of treating diabetes as well (Alkhalidy et al., 2018; Oza and Kulkarni, 2018; Wang et al., 2018; Xu et al., 2018; Wu et al., 2020; Matboli et al., 2021).

In the present study, we demonstrated that high glucose increased cell viability and proliferation, while HZQMY could reverse these phenomena. What’s more, since angiogenesis is a property of endothelial cells, the *in vitro* tubulogenesis experiment was carried out to observe the effect of HZQMY on HRCECs exposed to high glucose. As a result, tube formation of the HRCECs could be inhibited by HZQMY. In addition, we also found that HZQMY could increase the apoptosis of HRCECs, which is displayed as accelerating the nuclear pyknosis and cell late apoptosis.

**FIGURE 8:**
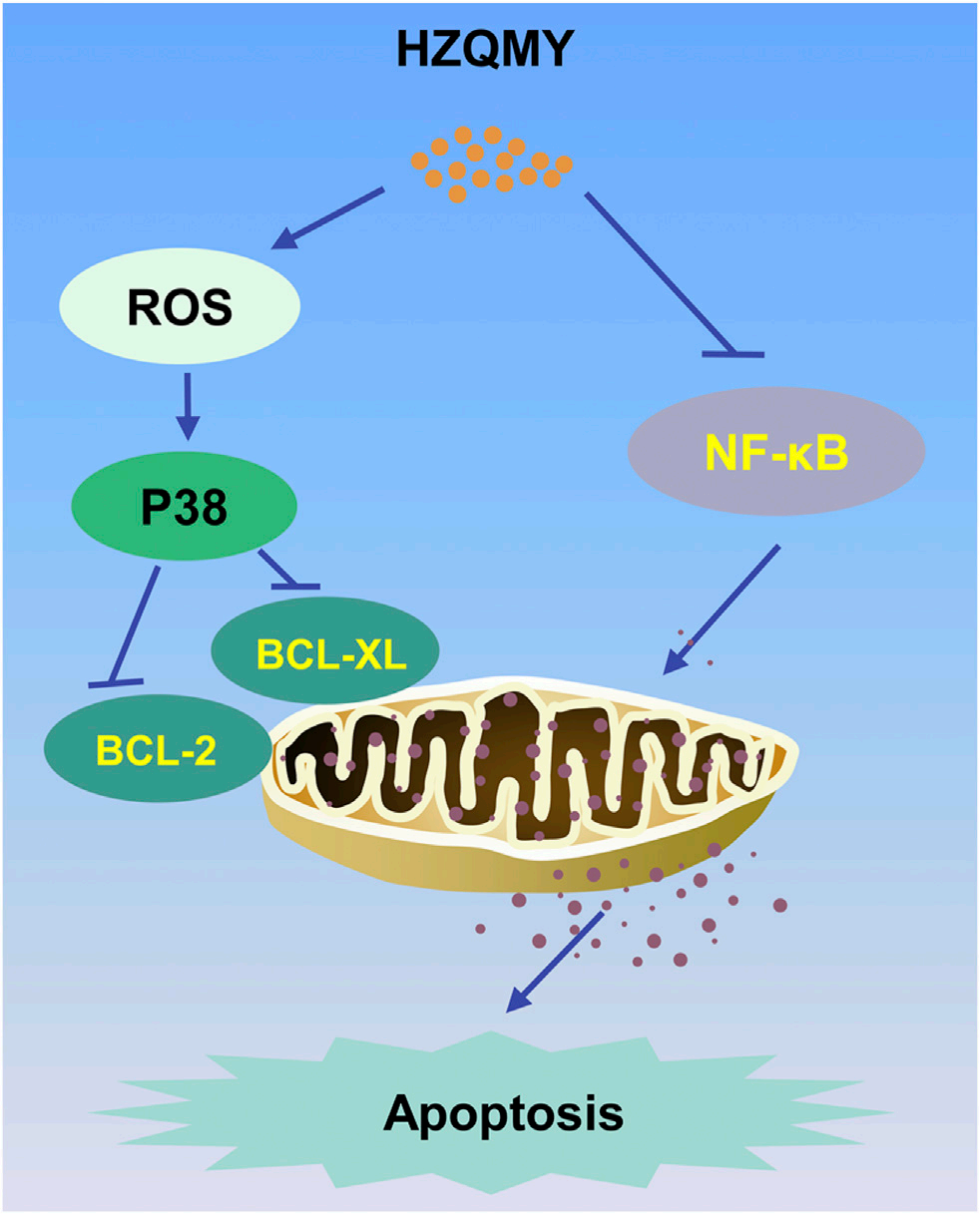
The mechanism of the inhibitory effect of HZQMY in HRCECs. HZQMY regulated ROS/P38 signaling pathway, and then inhibited the expression of BCL-2 and BCL-XL. What’s more, HZQMY also inhibited NF-κB signaling pathway to induce mitochondrial apoptosis.

We further explored the mechanism of action on the inhibition of cell proliferation and induction of cell apoptosis by HZQMY. The mitochondrial pathway is the most common apoptotic one (Ou et al., 2017). HZQMY was found to promote MMP collapse. Studies have indicated that promotion of P38 and ROS could promote mitochondrial apoptosis (Rao et al., 2012; Syed et al., 2020). Moreover, studies have also shown that ROS can regulate P38 induced mitochondrial apoptosis (Cui et al., 2019). Upon damage to endothelial cells, p38 is involved in the regulation of ROS mediated damage to endothelial cells (Usatyuk et al., 2003). In our study, HZQMY could increase p-P38 and ROS concentration and decrease ATP level. What’s more, HZQMY also downregulated the expression of BCL-XL and BCL-2 in HRCECs, indicating that HZQMY promoted cell apoptosis through the mitochondrial pathway. In addition, NF-κB regulates programmed cell death, cell adhesion, proliferation, innate and adaptive immune responses, and tissue remodeling (Ghosh and Hayden, 2008). Previous study showed that pantoprazole could promotes mitochondrial apoptosis by decreasing NF-κB signaling pathway (Geeviman et al., 2018). Our experiment also showed that high glucose increased the expression of p-NF-κB in HRCECs, while HZQMY could reverse their expression. Meanwhile, it was observed that the G_2_/M phase accounted for a higher proportion in the HZQMY group than in the HG group, indicating that HZQMY could regulate P38 and NF-κB pathway and interfere with the cell cycle, thereby inhibiting cell proliferation and promoting apoptosis (Figure 8).

With the rapid development of proteomics and the establishment of bioinformatics theory, problems such as low detection sensitivity and few reliable evaluation indexes for clarifying the mechanism of action of numerous active compounds from Chinese medicines have been gradually solved, and a new strategy has been provided for the research on the target of these compounds in the treatment of diseases. KEGG analysis showed that the differentially expression proteins were mainly enriched in apoptosis, and the signaling pathways involved are most likely to be HIF-1. What’s more, we also screened out 5 most likely target proteins through PPI analysis, which were HSPA4, MAPK3, ENO1, EEF2 and ERPS. High glucose combined with palmitate can regulate the expression of HSPA4 (Diaz-Ganete et al., 2021). MAPK3 (also known as ERK1) is necessary for pancreatic β cell function (Leduc et al., 2017). In diabetic patients, elevation of glycolytic enzymes including ENO1 is used as a biomarker for endogenous renoprotective factors (Gordin et al., 2019). Aerobic exercise combined with resistance exercise is able to enhance the activation of EEF2, contributing to improved glycemic control and maintenance of muscle health in type 1 diabetes (Minnock et al., 2020). ERPS can also be the potential biomarker to distinguish the type 2 diabetes and healthy person (Chu et al., 2013). What’s more, inhibiting ERK1/2-NF-κB pathway could attenuate inflammation of DR (Zhang et al., 2019). These results suggest that these are probably the key proteins of HZQMY acting on HRCECs exposed to high glucose, which need to be further studied.

In conclusion, HZQMY inhibited the proliferation and promoted the mitochondria related apoptosis of HRCECs under high glucose exposure possibly by regulating P38 and NF-κB pathway. The target proteins need to be verified further.

## Data Availability

The original contributions presented in the study are included in the article/supplementary material, further inquiries can be directed to the corresponding authors.
